# The role of procurement frameworks in responsible AI innovation in the National Health Service: a multi-stakeholder perspective

**DOI:** 10.3389/frhs.2025.1608087

**Published:** 2025-06-05

**Authors:** Thomas D. Evans, Omer Ahmad, Joseph E. Alderman, Georgia Bailey, Peter Bannister, Nick Barlow, Natalie Davison, Amanda Isaac, Aditya U. Kale, Trystan MacDonald, Qasim Malik, Susan C. Shelmerdine, Henry David Jeffry Hogg, Alastair K. Denniston

**Affiliations:** ^1^ AI and Digital Health Team, University of Birmingham, Birmingham, United Kingdom; ^2^ Special Care Dental Services, George Eliot Hospitals NHS Trust, Nuneaton, United Kingdom; ^3^ Division of Surgery & Interventional Science, University College London, London, United Kingdom; ^4^ College of Medicine and Health, University of Birmingham, Birmingham, United Kingdom; ^5^ National Institute for Health and Care Research (NIHR) Birmingham Biomedical Research Centre, Birmingham, United Kingdom; ^6^ University Hospitals Birmingham NHS Foundation Trust, Birmingham, United Kingdom; ^7^ Skin Analytics, London, United Kingdom; ^8^ Romilly Life Sciences, London, United Kingdom; ^9^ DrDoctor, London, United Kingdom; ^10^ School of Biomedical Imaging and Imaging Sciences, King’s College London, London, United Kingdom; ^11^ Royal College of Radiologists, London, United Kingdom; ^12^ Department of Ophthalmology, Queen Elizabeth Hospital Birmingham, University Hospitals Birmingham NHS Foundation Trust, Birmingham, United Kingdom; ^13^ Institute of Inflammation and Ageing, University of Birmingham, Birmingham, United Kingdom; ^14^ Respiratory Department, Birmingham Women’s and Children’s NHS Foundation Trust, Birmingham, United Kingdom; ^15^ Department of Clinical Radiology, Great Ormond Street Hospital for Children, London, United Kingdom; ^16^ UCL Great Ormond Street Institute of Child Health, Great Ormond Street Hospital for Children, London, United Kingdom; ^17^ NIHR Great Ormond Street Hospital Biomedical Research Centre, London, United Kingdom; ^18^ Department of Applied Health Research, School of Medical Sciences, College of Medicine and Health, University of Birmingham, Birmingham, United Kingdom; ^19^ Birmingham Health Partners Centre for Regulatory Science and Innovation, Birmingham, United Kingdom; ^20^ National Institute for Health and Care Research Biomedical Research Centre, Moorfields Eye Hospital/University College London, London, United Kingdom

**Keywords:** artificial intelligence, procurement, frameworks, National Health Service, implementation

## Abstract

Procurement carries legal requirements across public services in the UK but, for stakeholders in clinical Artificial Intelligence (AI) innovation, it is often poorly understood. This perspective piece summarises insights from a cross-sector workshop exploring the role of procurement frameworks in supporting AI innovation in the National Health Service (NHS). The significant characteristics of AI from a procurement perspective are identified and their consequences are explored. The workshop identified challenges including visibility of AI procurement processes, uncertainty in the value in AI products, process inefficiencies, sustainability and framework design. Opportunities relating to AI procurement were also identified. These insights highlight the potential for procurement frameworks to enable responsible AI innovation in healthcare but acknowledge the need for collaborative efforts from a range of stakeholders to overcome the difficulties experienced by many to date.

## 1 Introduction

Procurement is the act of buying products or services in a structured way to ensure best value for an organisation and compliance with relevant legislation. The UK Procurement Act 2023 requires that any high-value item or service purchased by a public service, including the NHS, is subject to a procurement exercise (1). Procurement is a process rather than just a purchase requiring input from vendor and buyer. Competing vendors are invited to bid (tender) for a contract and these bids are then evaluated and scored against minimum and desirable criteria. If only one vendor meets the minimum criteria, a contract can be directly awarded to them. Where multiple vendors meet these criteria bids are compared and the contract is then awarded to the vendor with the highest score.

The NHS is a complex network of interconnected organisations and much of the responsibility for procurement is delegated to different structural levels, e.g., Integrated Care Systems (ICS) or NHS trusts. There is also substantial variation between the devolved nations in the way in which central funding can be made available for local procurements. Instead of an allocated budget from NHS England, there are block grants provided for National Services Scotland (National Procurement in Scotland), NHS Shared Services System in Wales, and the Procurement and Logistics Service in Northern Ireland. This structure and variation means that vendors often must sell their products to multiple organisations with varying resource and procurement expertise. Framework agreements are a type of contract that reduce the burden of procurement for NHS buyers and reduce repetition for vendors selling their product(s) across the NHS. They are currently used for approximately £25 Billion of the £30 Billion annual total spent on third party products and services by the NHS (1). Framework agreements work by pre-approving suppliers who meet the minimum criteria, and then setting out the terms and conditions through which future purchases can be made. These framework agreements typically last for several years, and there are varied approaches to inviting new vendors to tender throughout that period. These include *fixed terms*, which are only open to application at their outset, *open frameworks* with periodic opportunities for application, and *Dynamic Purchasing Systems* (DPS) with relatively continuous opportunities for new applications from vendors. Each framework focuses on a specific scope of products and services and will typically contain several lots (subcategories of a framework) to curate products and services into narrower scopes, e.g., by clinical area.

Framework agreements are produced by Framework Hosts, organisations who receive commission for the contracts awarded through their frameworks. Following a recent move to consolidate the current range of over 1,200 frameworks, a new accreditation process has set out 20 framework hosts for use in the NHS (2). These hosts are aligned to 6 categories (termed pillars) of products and services, with a few preferred framework hosts identified for each pillar (3).

**Figure 1:**
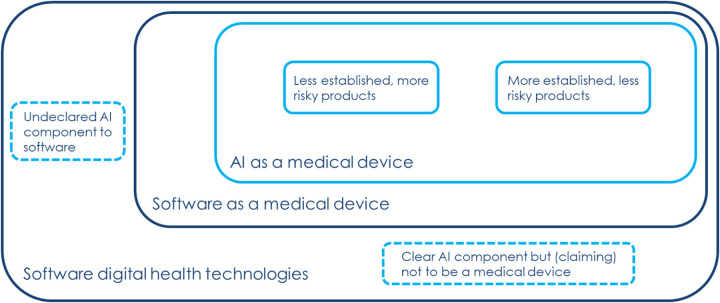
Overview of current landscape of software digital health technologies, outlined by one of the industry attendees.

Whilst the use of frameworks is well established in many areas, their application to emerging Artificial Intelligence (AI) products and services present new challenges and opportunities. A schematic laying out categories of digital health software, and AI in relation to those categories, is outlined in Figure 1. This approach to categorisation focuses on key characteristics of whether a digital health software meets regulatory definitions to qualify as a *medical device* and if it contains elements that meet technical definitions of AI (3). For vendors of AI products and services, successfully applying to a framework presents an important step toward implementation. AI products have sat on both software based framework agreements, as well as on AI only frameworks (4). In March 2022, the first health and AI specific procurement framework was released to target diagnostic imaging for stroke (5). Contracts made through this framework had central NHS funding, supporting complete uptake across the established stroke networks in England (6). However, this example was limited to a single clinical domain, and a continuation of this approach could precipitate a new proliferation of framework agreements.

The Incubator for AI and Digital Healthcare is an National Institute for Health Research (NIHR)-supported cross-sector community of practice to support safe and responsible innovation (7). A range of voices from within this community highlighted the perception of procurement as a barrier to AI innovation, and as an area which is poorly understood. It was also apparent that some of the more general challenges expressed by the community around evaluation and due diligence for AI may be partly addressed by procurement frameworks and their hosts. Drawing on this community, a cross-sector and interdisciplinary workshop was planned to surface and characterise current challenges and opportunities around the use of framework agreements in the procurement of AI products and services. Attendees spanned industry, procurement professionals, clinical, regulatory and technical teams. The workshop was designed as three sessions, with questions, comments and debate after each. This included a series of scene-setting short talks from key stakeholder groups, and two semi-structured small group discussions (see supplement for topic guide). This perspective piece summarises insights gained from the workshop drawn from the contemporaneous notes of facilitators and authors. It aims to guide AI vendor and NHS buyer organisations as they navigate procurement processes and provide evidence to framework hosts and policy makers as they influence the future landscape of AI procurement in the NHS.

## 2 Challenges

AI products have several characteristics that challenge traditional procurement by framework—these are outlined in Table 1. These challenges are presented below within the themes of visibility, value, process, sustainability and framework design.

**Table 1:** Characteristics of AI that can present challenges to procurement.

Characteristic	Challenge
Immature/young market	There are no clear market leaders and often a limited number of competitors in a particular field. Buyer experience of purchasing these tools is also limited
Multidisciplinary	Innovation in healthcare AI is reliant on the coordination of historically siloed disciplines with few examples of effective cross functional teams
Uncertain value	Few AI tools have evidence generated from real-world deployments and have unproven value. This uncertainty affects both vendors and buyers
Limited generalisability	An effective deployment does not guarantee success in different settings, or from competitor products applied for the same purpose
Revenue vs. capital spend	AI is often deployed as software as a service (SaaS) and is more suited to revenue rather than capital spend, which is typical for other software digital health technologies

### 2.1 Visibility

Many stakeholders are not aware of procurement processes, especially clinicians who are commonly the end-users and often drive AI implementation in clinical practice. Not only that, but the role of procurement is not always understood by other stakeholders, often leading to late involvement of procurement expertise introducing implementation delay or abandonment. This lack of visibility extends to vendors who lack clear guidance on how to sell AI products to the NHS network and may encounter unexpected additional requirements during procurement processes.

### 2.2 Value of AI

There is limited precedent to inform estimates of the value that AI products will return to buyers. AI is frequently justified on economic terms, but can fail to account for the largely unknown costs of implementation and infrastructure, and post-market surveillance (8). The value of AI is often context-dependent, and downstream from deployment, and can also require real-world deployment for value assessment. As a group of technologies, AI is susceptible to drift in performance over time meaning that even once value return is characterised it is likely to change. Thorough evaluation of AI products may require a formal Health Technology Assessments (HTA), which are beyond the scope of framework agreements, but have only been completed on a small proportion of products on market (9). Beyond economic value, procurement processes also consider social value, but opportunities for social value return are not often realised due to challenges in measurement and lower prioritisation in practice.

### 2.3 Process inefficiency

Information governance (IG), technical, cyber security, and other demands lead to duplicate and differing requests from different buyers. This occurs despite efforts by vendors to complete generalisable proformas as buyers often modify national proformas to seek assurance in line with local risk appetite, but do not share these modifications due to the perceived risk of appearing as an outlier among peer organisations with no clear AI-specific standards of good practice to point to. Vendors can be reluctant to disclose some of the information sought during the tender processes due to commercial sensitivity concerns, but buyers can perceive these claims of commercial sensitivity as evasive. Also of note is that Small and Medium Enterprises (SMEs), which represent most AI vendors, may struggle to meet the resource demands of procurement processes. Similarly, buyers’ procurement team resources are stretched by the changing procurement landscape, requiring teams to manage contracts under new and old procurement legislation and the requirements of a young and dynamic AI market.

### 2.4 Financial sustainability

Funding for healthcare AI has often been non-recurring, being purchased through time limited funded frameworks or short-term contracting. This creates financial uncertainty for buyers and a potential funding “cliff-edge” for vendors if multiple contracts close simultaneously. Not all adopters have the digital infrastructure or personnel required for AI innovations, meaning that more mature organisations benefit disproportionately, potentially widening inequalities between NHS organisations. With AI product vendors likely to be SMEs there is increased vulnerability to acquisitions, mergers and failures. Beyond the point of procurement, the ongoing resource implications of post-market surveillance are variable and poorly characterised, presenting unknown long-term costs which threatens the sustainability of AI use.

### 2.5 Framework design considerations

Framework characteristics impact upon their utility and use. For example, fewer frameworks and framework hosts increase the potential market share that each can leverage in price negotiations. Fewer framework hosts may also consolidate the limited expertise in AI evaluation and procurement across the system, and reduce complexity for buyers and vendors, aligning with wider NHS procurement strategy. Criticisms of AI-specific framework designs include that they may lead to proliferation of frameworks or could exclude suitable effective solutions without an AI component. This could encourage solution rather than needs-led innovation (i.e., overlooking effective but less complex solutions for AI) and exacerbate the complexity of the current procurement landscape. On the other hand, non-AI-specific frameworks may pose requirements that are inappropriate for AI solutions (8), excluding market contributions from otherwise relevant products. Any framework can exclude new-to-market vendors if application periods do not match vendors' timelines. This risk is particularly relevant to the AI market with new SME vendors emerging at a high rate. Dynamic Purchasing Systems or open frameworks may mitigate this risk but can be more costly to run for framework hosts who may adopt less flexible framework designs to manage their own internal resources.

## 3 Opportunities for improvement

Several suggestions were made throughout the workshop to address some of the challenges of AI procurement in the NHS, but also to AI innovation more generally. Realising these opportunities will require collaborative efforts from multiple stakeholder groups:

• Integrated and early involvement of procurement teams within cross-functional AI teams to reduce resource waste on unviable projects and improve buyers' implementation efficiency.
• Wider promotion of supplier registries (i.e., Atamis) to spread knowledge of what AI can be bought and how sales can be made to the NHS across stakeholder groups.
• Provide opportunities to draw on external expertise through communities of practice, peer collaboration or third-party suppliers offering specialist AI implementation teams.
• Leverage provisions of the 2023 Procurement Act such as the Provider Selection Regime and emphasis on Dynamic Purchasing Systems to facilitate timely and sustainable contracting opportunities (1).
• Design frameworks and lots around clinical problems rather than available technology types to prioritise product value proposition over novelty.
• Specification of key performance indicators within frameworks that establish the type and scale of value contracts are expected to deliver.
• Frameworks that hold vendor compliance documentation (e.g., Data Protection Impact Assessments, model cards, clinical evidence) providing documents which meet buyers' due diligence needs and enhance transparency (10).
• Tender requirements for AI that accommodate forms of social value aligned to patient and public needs, e.g., task shifting to improve patient care accessibility and staff role expansion, bias mitigation and environmental impact.

## 4 Discussion

This workshop confirms procurement's position as a key step in the clinical AI lifecycle, where the goals and expectations of different stakeholders must be reconciled and committed to contract. It surfaces challenges inherent to AI innovation; uncertainty over the form and scale of value on offer, complex and multifaceted risk for adopters, and a need for place-based evidence. It is not surprising that many stakeholders experience friction or even frustration at this mandatory crossing point of different perspectives and priorities. Some of this friction is necessary, representing the work of collective sense-making with cross-sector experts pooling their expertise to make informed decisions that serve all parties. The perspectives shared here make it clear that there are also opportunities to intervene at different levels, to remove unnecessary friction experienced around procurement. These interventions could also improve the efficiency and effectiveness of AI innovation in healthcare more broadly.

At the system level, interventions to simplify the procurement landscape and integrate commercial considerations within stakeholders' understanding of the innovation pipeline are already underway in the UK. One example is the Integrated Rules Based Pathway for MedTech, aiming to communicate NHS needs and approaches to measuring value for digital innovation as products are developed and provide practical details over how mature products can be sourced and procured if they receive positive health technology assessments (11). Furthermore framework agreements have already been used as a mechanism to reduce friction in the uptake of AI technologies, with the example of a funded framework resulting in 100% uptake across the 107 NHS stroke centres in England (5, 6). Another example is the 2023 Procurement Act, which should enable more dynamic approaches to procurement that accommodate the needs of fast-moving markets including clinical AI. At an organisational level, the establishment of cross-functional AI teams can bring together the collective skills and experience required to identify, analyse and manage the varied risks and benefits of AI innovation in a coordinated fashion (1). Exemplar cross-functional AI teams are emerging across the NHS and should be encouraged, with procurement embedded to prevent misdirected resource investment in the innovation process (12). At an individual level, wider practical awareness of procurement principles and tools such as frameworks can also help to align the efforts of stakeholders without procurement expertise across the ecosystem. Educational offerings to realise this opportunity are available, but widening access and uptake could yield greater benefits.

Acknowledging the early stage of the market for clinical AI, procurement processes agreements will continue to play an important role alongside frameworks, for the buyers with expertise and resource to pursue them. However, appropriately designed procurement frameworks can serve to catalyse each of the system, organisational and individual level interventions described above. They have the potential to elevate assured AI products and services, clearly signposting the evidence base and rationale for their use in the NHS. Refining procurement frameworks and fostering multi-stakeholder engagement, the NHS could not only mitigate friction in AI adoption but also accelerate the responsible and effective integration of AI innovations into clinical practice, ultimately enhancing patient outcomes and system-wide efficiencies.

## Data Availability

The original contributions presented in the study are included in the article/Supplementary Material, further inquiries can be directed to the corresponding author.
