# Supplementary material for: The role of procurement frameworks in responsible AI innovation in the National Health Service: a multi-stakeholder perspective
Source: Front Health Serv. 2025 Jun 5;5:1608087. doi: 10.3389/frhs.2025.1608087 (PMC12176890; doi:10.3389/frhs.2025.1608087)
Supplement: Supplementary file 1 [file Supplementaryfile1.pdf]

Supplementary materials

S1 – Topic Guide

Session 2 -What challenges does NHS procurement currently present for AI innovation and what opportunities do frameworks present?

1a Value for money

- Can anyone share experiences of price negotiation for AI procurement in the NHS?
- Do we think prices vary for the same product between trusts?
- Are there generalisable forms of value we should expect from AI technologies? (e.g. time saving, improved diagnostic accuracy, financial saving etc.)
- Should prices for AI products be use-related or at a flat monthly rate?

1b Ease of market access

- What challenges do procurement frameworks present selling to the NHS?
- How do procurement frameworks make selling easier?
- Do current procurement processes favour certain types of AI products or vendors over others? (e.g. is it accessible to SMEs)
- Are there any changes to the way frameworks are designed or disseminated that could help AI product or service suppliers access NHS buyers?

1c Due diligence

- Are there common questions that buyers ask of vendors that could be answered at the framework application stage (e.g. IUS, data flows, clinical evidence, clinical risk file, PMS, underlying technology/architecture)?
- Are there any requirements for all or some AI products and services that should be demanded beyond the current list? (regulatory compliance, DTAC, interoperability standards, cloud security principles etc. – see briefing doc)
- Are there assurances specific to AI health technologies that have not sought regulatory approval as medical devices that should be considered (e.g. ambient voice recording)?
- Are any requirements on the current list problematic? (e.g. ICD10 conformant)
- Are there any commercial sensitivities around information we would like vendors to share with framework hosts and buyers?
- Are there any mitigations we could suggest for these commercial sensitivities?

1d Social value

- Which of the 5 social value objectives seem most relevant to AI products and services (Covid-19, Financial wellbeing, Fighting climate change, Equal opportunity)?
- What specific examples of AI-enabled social value come to mind within these definitions/scopes?
- What evidence of those forms or value would be meaningful and reasonable to ask for?
- Should the expectation or focus differ between types of AI products and services?

1e Framework accessibility

- How easy is it to understand what frameworks relevant to AI are available and what products and services are eligible or listed?
- What limits understanding of frameworks for different stakeholders at the moment and how might that be improved?
- What might the impact be of improving awareness of procurement frameworks among different stakeholders in NHS AI innovation?
- Are there reasons to limit the transparency of frameworks, lots or listed products and services?

Session 3 - How should procurement frameworks for AI products and services be designed and disseminated?

2a Scope of AI frameworks

- Should AI products and services be listed on the frameworks that include other types of software?
- How important are dynamic procurement systems for AI products and services as opposed to frameworks with single fixed timepoints for application?
- What products and/or services should be included on AI framework(s) (AI as a medical device, AI in a software device, non-medical device AI health technologies, adopter consultancy services)?
- Should clinical (e.g. AIaMD) and non-clinical AI products (e.g. scheduling tools) and services be included in the same framework?

2b Number of frameworks and hosts

- What are the advantages of limiting AI products and services to one (or a few) procurement frameworks?
- What are the advantages of encouraging many frameworks to be developed for AI products and services?
- What are the advantages of limiting AI products and service frameworks to one (or a few) frameworks hosts?
- What are the advantages of encouraging many framework hosts to develop AI products and services?

2c Lot structure

- How should AI products and services be grouped into lots within a framework? (e.g. clinical specialty, regulatory classification, input modality, use case)
- Do different approaches to lot structure favour some stakeholders over others?
- Should services relevant to AI be included in lot(s) on AI frameworks? (e.g. implementation consultancy or AI research tools)

2d Type and level of evidence required

What kind of evidence and/or measure should be asked for in tendering documents regarding:

- Clinical risk – e.g. performance and post market surveillance
- Financial risk – e.g. return on investment, implementation costs
- Cyber risk – e.g. cyber essentials, details of cloud environment
- Information governance risk, e.g. data flows, ICO registration

- Technical feasibility/risk, e.g. interoperability, training and education

2e Stakeholder groups to target

- Which stakeholder groups is AI procurement frameworks be relevant to?
- Who could dissemination or education about AI procurement frameworks target to most effectively impact AI innovation in the NHS?
- What are the knowledge gaps that should be prioritised for this/these stakeholder group(s)?
- What kind of dissemination or education interventions might best achieve this? (e.g. professional networks/bodies to target, platform on which frameworks are hosted, degree of transparency over frameworks)
